# O084: Implementing a hand hygiene programme in the critical care department of galway university hospitals, Ireland: an interesting and challenging journey

**DOI:** 10.1186/2047-2994-2-S1-O84

**Published:** 2013-06-20

**Authors:** TW Boo, J Davitt, C Greally, M Commane, B Hanahoe, T van der Kooi, J Bates

**Affiliations:** 1 Bacteriology, School of Medicine, NUI, Galway, Ireland; 2 Clinical Microbiology, Galway University Hospitals (GUH), Galway, Ireland; 3 Infection Control, GUH, Galway, Ireland; 4 Critical Care, GUH, Galway, Ireland; 5 Clinical Microbiology, GUH, Galway, Ireland; 6 RIVM, Bilthoven, The Netherlands

## Introduction

As a participant of the PROHIBIT (Prevention of Hospital Infections by Intervention and Training) study, we review interim outcomes and practical challenges after 18 months of implementing a hand hygiene programme in our critical care units.

## Methods

Following a 6-month baseline audit, a multifaceted programme was instituted in July 2011. Regular audits tracked the programme’s impact on outcomes. Factors impacting on the programme’s implementation were collated from stakeholders’ feedback and observations made by the implementation team. These factors were then used to further inform and adapt unit based interventions.

## Results

There was a significant decrease in central venous catheter-related bloodstream infection (CRBSI) rates following the programme implementation. CRBSI rates fell from 5.4 infections / 1,000 CVC days (95% confidence interval (CI) 3.1 – 9.5) in the baseline period to 0.8 (95% CI 0.3 – 2.1) during the 18 months of the programme. Hand hygiene compliance rates rose from 48.8% (baseline period) to 77.2% during the period of programme implementation, with results maintaining above 75% in the latter 12 months (Jan – Dec 2012). Factors facilitating itsimplementation include institutional endorsement of outcome measures as key performance indicators, regular feedback to stakeholders, targeted educational sessions, and bedside shadowing exercises. Challenges have also been encountered, eg. maintaining motivation and enthusiasm of staff, waning of the ‘novelty’ factor in the study, maintaining hand hygiene as a priority in challenging times, poorer compliance rates of visiting medical teams.

## Conclusion

Participation in the PROHIBIT study gave us the impetus to implement an intensive hand hygiene programme in our critical care units. Although resource-intensive, it has been a success to date. The journey to improve hand hygiene compliance has also been one about shared vision and culture change. Our journey continues.

## Disclosure of interest

None declared.

